# Preventive Dentistry

**Published:** 1916-09

**Authors:** 


### PREVENTIVE DENTISTRY.

A breezy, helpful little article occurs in *Dental Review* (June) by Dr. St. A. Chamberlain on methods to be employed in preventing dental ailments. The author, in reviewing briefly modern methods of dentistry, shows how it is possible, even under the best conditions, to cause areas about the tooth that will be possible source of subsequent infections. He holds to the view that the best dentistry is that which will prevent the loss of tooth tissue.

Taking into consideration the methods usually employed, the author shows how easy it is to bring undesirable conditions. In devitalization, if arsenic is used, the periapical tissues is attacked, its vitality is lowered, and it becomes susceptible to infection through the blood stream. If pressure anesthesia is used there is danger of infective material being forced through the apex. When treating putrescent root canals with strong drugs, the apical pericemental tissues suffer. The operation of filling root canals, unless free from sources of infection, will cause new troubles to occur later on. Crown and bridge work, either on vital or devitalized teeth, is attended with grave danger to the subject. In fact, no operation in dentistry, having in view the restoration of lost tissue, is above criticism. The best dentistry, then, is preventive rather than restorative in character.

Now for the author's suggestions for the prevention of dental ailments. First, the patient is instructed as to a course of home treatment. He is advised "to equip himself with a proper tooth brush, one with the bristles arranged in tufts, and the length of the bristles graduating from the short bristles at the end to long ones near the handle. Inasmuch as it is the end of the bristle which does the cleaning, none of them should be so long that they bend while sliding the brush over the teeth. Start the brush on the gums and with a rolling motion sweep down over the teeth." Young patients are instructed to use dental floss and older patients to use the flat floss for polishing between the teeth. A saponaceous tooth paste is to be used and followed by a vigorous rinsing of the mouth with lime water. In extreme cases of caries and denuded roots the mouth cavity is to be swabbed with a solution of Iodo-glycerol. "Iodin has great penetrating properties as well as germicidal properties, and will penetrate the mucous plaques as well as deposits of fermenting foods."

When the patient visits the office for treatment the first thing to do is to remove all calcareous deposits above and below the gingiva. The surfaces of the tooth are then polished with flower of pumice, using dental engine with cups and disks. Flat floss is used to polish the prominent surfaces "not only pass floss between the teeth, but polish the proximal surfaces carefully, flooding the field of operation. Finish and spray, using compressed air."

An abrasive, applied with engine polishing brushes, is then used on the sulci and pits. After drying the tooth, the pits and fissures are flooded with twenty per cent. Ag. No. 3 and worked in with a sharp explorer.

"This should be dried, and either white or black copper cement worked into all the crevices, leaving as much on the occlusal surface of the tooth as is possible without interfering with the occlusion. This cement will gradually wear away, leaving the fine lines still sealing the weak places for a long period of time, when the same can be repeated."—*Oral Health.*

